# Within-host diversity of MRSA antimicrobial resistances

**DOI:** 10.1093/jac/dkv119

**Published:** 2015-05-08

**Authors:** Kinga I. Stanczak-Mrozek, Anusha Manne, Gwenan M. Knight, Katherine Gould, Adam A. Witney, Jodi A. Lindsay

**Affiliations:** 1 Institute for Infection and Immunity, St George's, University of London, Cranmer Terrace, London SW17 0RE, UK; 2 Centre for Mathematical Modelling of Infectious Diseases, London School of Hygiene and Tropical Medicine, Keppel Street, London WC1E 7HT, UK

**Keywords:** transduction, mobile genetic elements, horizontal gene transfer, whole-genome sequencing

## Abstract

**Objectives:**

MRSA is a major antimicrobial resistance (AMR) pathogen. The reservoir of infecting isolates is colonization, which is the site of evolutionary selection. The aim was to identify if AMRs in colonizing MRSA populations diversified and potential mechanisms of resistance gene transfer *in vivo*.

**Methods:**

Nasal swabs from 38 MRSA carriers admitted to hospital were plated and 20 individual colonies from each patient tested for phenotypic antibiotic susceptibility and genetically for lineage, carriage of four prophages and three plasmid families. Free bacteriophages were detected in swabs as well as their capacity for transducing resistance genes.

**Results:**

Nine (24%) patients carried phenotypic AMR variants and 24 (63%) carried prophage and plasmid variants. If a single colony was selected for testing, the probability of detecting all AMR in that patient was 87%. Sixty-four different AMR and mobile genetic element (MGE) profiles were detected, mostly in the MRSA CC22 background (where CC stands for clonal complex), with up to 8 profiles per patient. Nearly half of the patients carried detectable free bacteriophages and phages successfully transduced resistance genes between laboratory and patient isolates *in vitro*. WGS showed MRSA core genomes were stable, while AMR and MGEs varied.

**Conclusions:**

‘Clouds’ of MRSA variants that have acquired or lost AMR and MGEs are common in nasal colonizing populations and bacteriophages may play an important role in gene transfer. Accurate estimation of AMR and genetic variability has implications for diagnostics, epidemiology, antimicrobial stewardship and understanding the evolutionary selection of AMR in colonizing populations.

## Introduction

Antimicrobial resistance (AMR) risks the future of modern medicine and one of the most problematic pathogens globally is MRSA.^1,2^ Resistance to all classes of antimicrobials has been detected in MRSA and the majority are due to resistance genes encoded on mobile genetic elements (MGEs) such as plasmids and transposons.^3,4^ Despite the prevalence of these resistances, they do not appear to be accumulating into fully drug-resistant clones. Instead, epidemiological evidence suggests resistances may transfer frequently and may also be lost frequently.^5,6^ However, little is known about AMR transfer in the host and the factors that control transfer, selection or deselection of resistances in MRSA. To reduce AMR pathogens globally, we must understand the evolutionary pressures acting on colonizing pathogens and develop strategies to reduce AMR selection.

The reservoir of infecting isolates is colonization in the nose.^7–9^ In a recent study of *Staphylococcus aureus* colonization in gnotobiotic piglets, extremely high levels of horizontal gene transfer (HGT) and loss of MGEs were detected.^10^ In particular, bacteriophages and plasmids transferred, including resistance plasmids, despite no exposure to antimicrobials. Loss of transferred elements was also common and individual piglets carried unique populations despite close contact. Mixed populations with a variety of MGE profiles were maintained during the course of the 16 day experiment, rather than selection of the fittest variant. HGT and loss during piglet co-colonization was significantly more common than during *in vitro* culture.^10^

Whether this variation occurs during human colonization is unclear. Although most humans are colonized with a single clone,^11–13^ recent studies have suggested ‘clouds’ of clonal variants can be detected. Specifically, Harris *et al*.^14^ showed that a colonized staff member in a neonatal intensive care unit that was the suspected reservoir of an outbreak carried variants with four to five SNPs and variation in an erythromycin resistance plasmid. Tong *et al*.^15^ showed that a colonized patient in a resource-poor hospital setting that was the likely reservoir of hospital outbreaks carried variants with 147 SNPs over 9 weeks. Golubchik *et al*.^16^ showed that for 13 colonized patients where primary plate colonies were pooled and frozen, 8–12 subcultured colonies showed variation in SNPs and MGEs. Similarly, in chronically infected cystic fibrosis patients, Goerke *et al*.^17^ showed that bacteriophages moved into and out of *S. aureus* populations over time. Chronic cystic fibrosis infection with *Pseudomonas aeruginosa* is also known to lead to diversification in bacteriophage and AMR profiles.^18–20^

The aim of this study was to investigate phenotypic AMR variation in MRSA colonizing populations of humans, as well as assessing genotypic variation in MGEs and potential mechanisms of gene transfer. Thirty-eight patients admitted to St George's NHS Healthcare Trust who were colonized with MRSA were included and 20 individual colonies from their primary nasal swab plates were assessed. We found evidence of frequent AMR gene transfer and even higher levels of MGE transfer. Generalized transducing phages capable of transferring AMR genes were prevalent in swab material.

## Materials and methods

### Sample selection and bacterial culture

Anterior nasal swabs from patients admitted to St George's Hospital (London, UK) were routinely plated onto mannitol salt agar plates containing 2 mg/L oxacillin (Sigma–Aldrich) and incubated for 48 h at 37°C to identify MRSA. Twenty-two MRSA-positive plates and their swabs were collected between February and April 2012 from carriers positive for nares culture and negative for groin culture; 16 swabs were collected from January to March 2013 from patients positive for nares culture (colonization of groin unknown). From each positive plate, 20 well-separated colonies (which will be referred to as subisolates) were selected at random and replated separately onto brain heart infusion agar (BHIA; Sigma–Aldrich) and incubated for 24 h at 37°C. Each subisolate obtained from BHIA plates was suspended in 1 mL of sterilized brain heart infusion broth (BHIB; Sigma–Aldrich) supplemented with 20% glycerol (Sigma–Aldrich), frozen and stored at −80°C until further analysis.

### Antibiotic susceptibility testing and AMR profiles

For each subisolate, susceptibility to 13 antibiotics was determined by the disc diffusion test in accordance with BSAC guideline version 11.^21^ An AMR profile is the combination of all resistances carried in a single isolate.

### Mathematical analysis

To estimate the probability of detecting all resistances in a discrete number of colonies, we used the distribution of AMR profiles from our 38 patients and 20 subisolates to run a simulation (Table S1, available as Supplementary data at *JAC* Online). This simulation assumed the same population of patients, with the same AMR profile distribution, and then randomly sampled one colony from each patient and recorded whether all resistances were seen. This was repeated 10 000 times in order to generate a probability of seeing all resistances (equal to the number of simulations where all AMR profiles were seen out of the 10 000). The same simulation was performed with two colonies picked from each patient, three colonies etc. up to a maximum of 100. We also analytically calculated the probability of seeing all resistances assuming that all patients had only one or two AMR profiles (using data from a subset of 37/38 of our patients).

### DNA extraction and amplification

Total genomic DNA from each single subisolate was extracted using the bacterial genomic DNA purification kit PurElute (Edge Biosystems) and 2.5 μL of lysostaphin (Sigma–Aldrich) was added. The restriction modification (RM) test was used to identify MRSA lineages as CC22, CC8, CC1 or CC45 (where CC stands for clonal complex).^22^ PCR was used to detect four major phage families based on their integrase gene (Φ1, Φ2, Φ3 and Φ6),^23^ two types of plasmid based on the replication locus (*rep*_10_ and *rep*_20_)^3^ and the putative antiseptic resistance gene (*qacA*) typically found on large plasmids, as these have previously been detected in MRSA CC22 populations.^3^ PCR was also used to screen for the presence of *tra* genes, necessary for conjugative transfer.^3^ All primers are listed in Table S2.

### WGS

Five subisolates from Patient 19 were selected for WGS. Patient 19 had two AMR and three MGE profiles (Table 1), and one subisolate with each profile (three subisolates) and two additional random subisolates from Patient 19 were chosen. Two sequenced subisolates (19A and 19B) were used in a transduction experiment as a recipient and donor (see below) and two random progeny cells were also sequenced.

**Table 1.** AMR resistance, genetic variability and free phages detected in 38 MRSA carriers

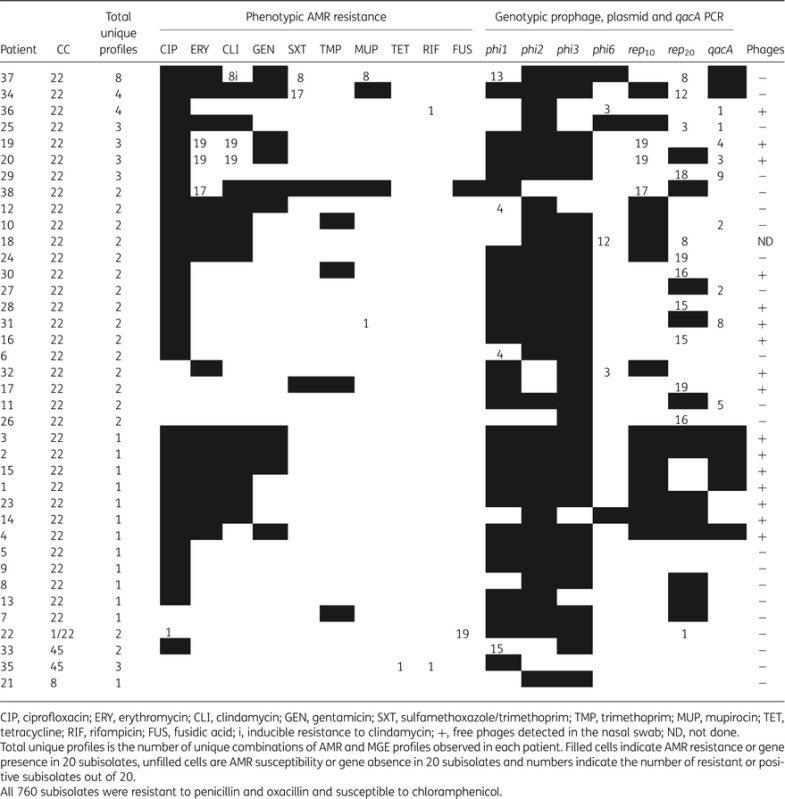

WGS was performed using the Ion Torrent™ Personal Genome Machine (Ion PGM™) in conjunction with Ion Torrent™ workflow reagents (Life Technologies, Paisley, UK). Genomic DNA (100 ng) was fragmented by sonication using a BioRuptor UD-200 (Diagenode, Belgium) and a library prepared using the Ion Plus Fragment Library Kit according to the manufacturer's instructions. The fragmented DNA was end-repaired, ligated to Ion-compatible adapters and nick-translated. To produce a median fragment size of ∼330 bp, each library was size-selected using a 2% E-Gel SizeSelect Agarose Gel (Life Technologies) and subjected to qPCR (Ion Library Quantitation Kit) for quantification. Five barcoded libraries were pooled in equimolar amounts and clonally amplified on Ion Sphere Particles™ (ISP) using the Ion OneTouch instrument. The template-positive ISPs were enriched using the Ion OneTouch ES and sequenced on the Ion 316 chip using a 200 bp sequencing kit. Sequence quality was assessed using Fastqc (http://www.bioinformatics.babraham.ac.uk/projects/fastqc/). Sequence assembly was performed using MIRA v3.9.17^24^ with default parameters for Ion Torrent data. Manual analysis and sequence inspection were performed using the Artemis^25^ and ACT^26^ genome visualization tools. SNP identification and phylogenetic reconstructions were performed as previously described, except that the reference genome was CC22 isolate HO 5096 0412 (accession number HE681097).^27^ Circos^28^ was used as a genome visualization tool to enable the identification and analysis of similarities and differences arising from genome comparisons. Contigs containing differences between genomes were analysed by BLAST^29,30^ to further ascertain gene content. If the contigs were similar to plasmid sequences in GenBank, then these contigs were searched by BLAST in an attempt to find reads that can circularize the plasmid.

### Phage isolation and titration

The bacteriophage isolation assay was modified from Aswani *et al.*^31^ Each original nasal swab stored in transport medium (Nuova Aptaca) was placed into a 15 mL Falcon tube containing 2–3 mL of phage buffer (50 mM Tris-HCl pH 7.8, 100 mM NaCl, 1 mM MgSO_4_, 4 mM CaCl_2_ and 1 g/L gelatin; Sigma–Aldrich). Suspended swabs were vortexed for 1 min and incubated for 2 h at 37°C at a shaking speed of 200 rpm. After incubation, the supernatant was filtered through a 0.22 μm filter (Sigma–Aldrich) to remove the bacterial cells and stored at 4°C.

Isolated phages were grown on RN4220 strain, centrifuged for 10 min at 4000 rpm and filtered through a 0.22 μm filter. The presence of phages was confirmed by the observation of clear phage plaques using the double-layer agar (DLA) technique. Briefly, 200 μL of phage mixture was mixed with 200 μL of recipient cells (RN4220 or 19B) in log phase (OD = 1 at *A*_600_) followed by addition of 30 μL of 1 M CaCl_2_. Samples were left to rest at room temperature for 15 min, mixed with ∼7 mL of top agar molten to 50°C and poured over set phage bottom agar plates. Phage agar was prepared by mixing 3 g/L yeast extract (Sigma–Aldrich), 3 g/L casamino acids (Fisher Scientific), 5.9 g/L NaCl (Sigma–Aldrich) and either 10 g/L agar (Sigma–Aldrich) (bottom) or 3.3 g/L agar (top). Plates were incubated at 30°C for 24 h and the number of plaques counted to calculate the pfu/mL of lysates.

From the DLA plates, two well-separated plaques were chosen for further analysis, one from Patient 19 (called Φ19) and one from Patient 20 (Φ20). Plaques were excised, resuspended in 2 mL of phage buffer and filtered through a 0.22 μm filter. Next, 100 μL of phage solution was added to mid-log-phase RN4220 resuspended in 7 mL of phage buffer, mixed with 7 mL of BHIB and incubated at 30–32°C at 70 rpm overnight. Lysates were centrifuged for 10 min at 4000 rpm, filtered through a 0.22 μm filter and stored at 4°C.

### Generalized transduction

RN4220 was used as a phage-negative recipient strain and strain KS*ermB* was constructed as a phage-negative donor strain in the RN4220 background (Table S3). RN4220 was chosen as it is RM deficient and able to accept DNA from the CC22 lineage used in this study. Two isolates from Patient 19, either erythromycin resistant (19A) or susceptible (19B), were also chosen as a donor and a recipient, respectively. Strain KS*ermB* was constructed by electroporation of *S. aureus* RN4220 by plasmid pCN50::*ermB*, constructed by cloning the entire *erm*(B) gene from *S. aureus* N315 into pCN50 and transforming into *Escherichia coli* DH5α. Briefly, purified plasmid was electroporated^32^ into RN4220 in the presence of 0.5 M sucrose (Sigma–Aldrich) and a subinhibitory concentration of erythromycin (0.15 mg/L; Sigma–Aldrich), as recommended by Brückner,^33^ incubated at 37°C for 2–3 h and then plated out onto BHIA supplemented with 0.5 M sucrose and 30 mg/L erythromycin and incubated for 24–48 h. The presence of *erm*(B) was confirmed by PCR.

To prepare lysates for transduction, donor strains (KS*ermB* or 19A) resistant to erythromycin were grown at 37°C with shaking at 80 rpm until log phase (OD = 0.5–1 at *A*_600_). Bacteria were spun down and resuspended in 7 mL of phage buffer plus 7 mL of BHIB. Filtered Φ80α phages or phages isolated from swabs (Φ19 and Φ20) were added, mixed gently and incubated at room temperature for 10 min. Tubes were placed into a waterbath and incubated at 30–32°C with shaking at 80 rpm. They were checked every 2 h and mixed gently. If after 6 h of incubation the visible effect of clearance was not observed, then samples were left overnight. After overnight incubation, samples were centrifuged for 10 min at 4000 rpm and filtered through a 0.22 μm filter.

The transduction assay was modified from Varga *et al.*^34^ and all strains used were negative for the presence of *tra* genes by PCR. Recipient bacteria (RN4220 or 19B) were grown in BHIB overnight at 37°C with shaking. After incubation, the bacterial culture was centrifuged for 10 min at 4000 rpm and resuspended in 1 mL of LK broth (1% tryptone, 0.5% yeast extract and 0.7% KCl; Sigma–Aldrich). Recipient cells were mixed with 1 mL of LK broth, phage lysate and CaCl_2_ (added to a final concentration of 8 mM). Samples were incubated at 31°C for 45 min. Control tubes with recipient cells only and phage lysate only were also prepared. After incubation, ice-cold 0.02 M sodium citrate was added (Honeywell International) to a final concentration of 15 mM and samples centrifuged at 4000 rpm for 10 min. The supernatant was decanted and the pellet resuspended in 1 mL of ice-cold 0.02 M sodium citrate and left on ice for ≥2 h. Samples were spread onto LK plates prepared by mixing LK broth components with 5 g of bacteriological agar supplemented with 0.05% sodium citrate and 0.15 μg/mL erythromycin, incubated at 37°C for 60 min and then overlaid with 4–5 mL of LK top agar supplemented with 30 mg/L erythromycin. Plates were incubated for 48 h at 37°C. The number of transductant cells was counted and expressed as the number of transductant cells/mL, instead of the commonly used frequency of transduction (pfu/cfu), as not all particles will carry virulent phage genome and cause lysis. The same experiments were performed using lysates made without addition of exogenous bacteriophages (donor cells only). To confirm that transductant cells did not result from transformation, each experiment was repeated in the presence of 20 μg/mL DNase (Promega). All transductant colonies were picked and passaged on mannitol salt agar and BHIA supplemented with 30 mg/L erythromycin. DNA from transductant cells was extracted and the presence of *erm*(C) or *erm*(B) confirmed by PCR.

### Statistical analysis

Fisher's exact test was used to compare the presence of phages or detected variation in patient populations from swabs collected in 2012 versus those in 2013. Differences in phage titres or transduction frequency were analysed by the unpaired Student's *t*-test and differences were considered statistically significant at *P* < 0.05.

## Results

### AMR differences within each patient

The majority of patients (89.5%) were colonized by subisolates belonging to the common UK healthcare-associated (HA)-MRSA lineage CC22 (Table 1).^5,35^ Two patients (5.3%) were colonized by MRSA CC45 lineage and one patient by MRSA CC8 (2.6%). Only one patient (2.6%) was found to be colonized by subisolates belonging to two different lineages (CC1 and CC22).

**Figure 1.**
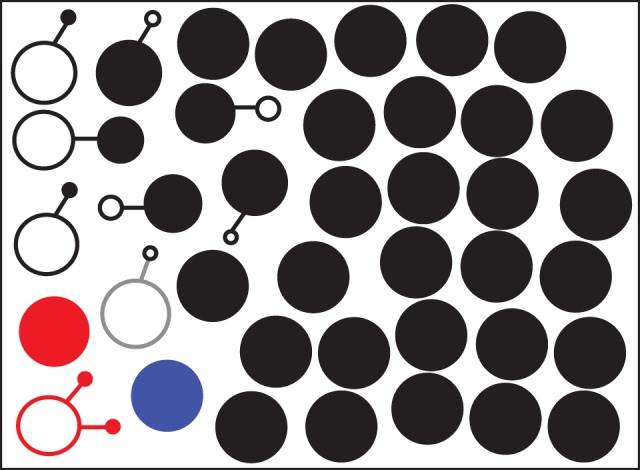
Phenotypic AMR profiles in 38 MRSA carriers. Each circle represents an AMR profile and connected circles are different AMR profiles from the same carrier. Circle size is proportional to AMR profile incidence in each patient and filled circles represent the AMR profile with the most resistances in that carrier. Black is lineage CC22, blue is lineage CC8, red is lineage CC45 and grey is lineage CC1. This figure appears in colour in the online version of *JAC* and in black and white in the print version of *JAC*.

Nine (23.7%) of the 38 patients were colonized with phenotypically distinct MRSA subisolates (Figure 1 and Table 1). Of these, five patients (Patients 19, 20, 22, 31 and 36) carried only one subisolate of 20 with a divergent AMR profile, one patient (Patient 35) carried two subisolates of 20 that differed from each other as well as differing from the remaining eighteen subisolates (these isolates were CC45, and are red in Figure 1), two patients (Patients 34 and 38) carried three subisolates with a divergent AMR profile, and one patient (Patient 37) carried eight subisolates with the same divergent AMR profile. Figure 1 also indicates the prevalence of AMR profiles carrying the most resistances (filled). In four patients with AMR variation, the most prevalent AMR profile also carried the highest number of resistances, but in four cases the most prevalent AMR profile did not carry the most resistances, and in one case the two AMR profiles had the same number of resistances (CC1; Figure 1 in grey). There was no significant difference in the proportion of patients showing phenotypic variation between samples collected in 2012 (Patients 1 to 22) and those collected in 2013 by Fisher's exact test.

An extensive range of variant AMR profiles were detected within the CC22 MRSA clonal population, suggesting the acquisition and loss of AMR genes is frequent, consistent with previous studies.^5,9^ Overall, all subisolates were resistant to penicillin and oxacillin, which is expected as the primary plates contained oxacillin, 89% were resistant to ciprofloxacin, 26% were resistant to gentamicin, 46.7% were resistant to erythromycin, 40.3% were resistant to clindamycin, 8.5% were resistant to co-trimoxazole, 13.2% were resistant to trimethoprim, 6.4% were resistant to mupirocin, 0.13% were resistant to tetracycline, 0.26% were resistant to rifampicin and 5.13% were resistant to fusidic acid. Resistance to chloramphenicol was not detected.

### Choosing colonies for AMR analysis

**Figure 2.**
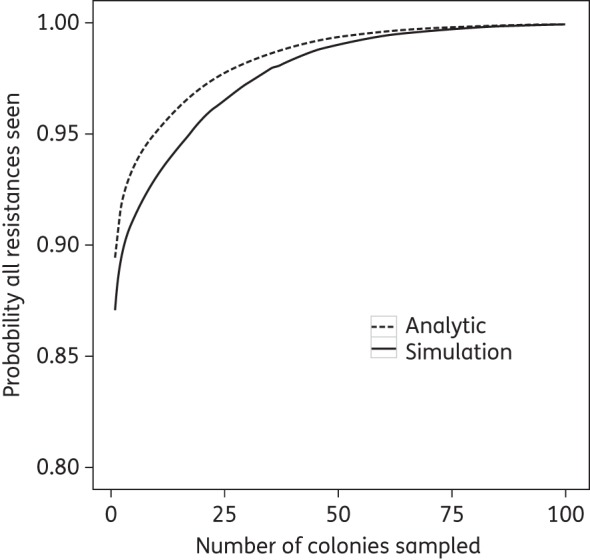
Probability of seeing all resistances in the patient by number of colonies sampled. The analytic solution has a higher probability as it assumes each patient only has up to two AMR profiles, whilst the simulation output (from 10 000 simulations) includes a patient with three profiles.

From our simulations, sampling 18 colonies is likely to give a 95% probability of detecting all resistances present in the sample (Figure 2). This would rise to 99% only if 50 colonies were sampled. If only one or two AMR profiles were found in all patients (as was the case for 37/38 of our patients), the analytical analysis showed that sampling eight colonies would give a 95% probability of detecting all resistances (Figure 2).

### Genetic variation within each patient

**Figure 3.**
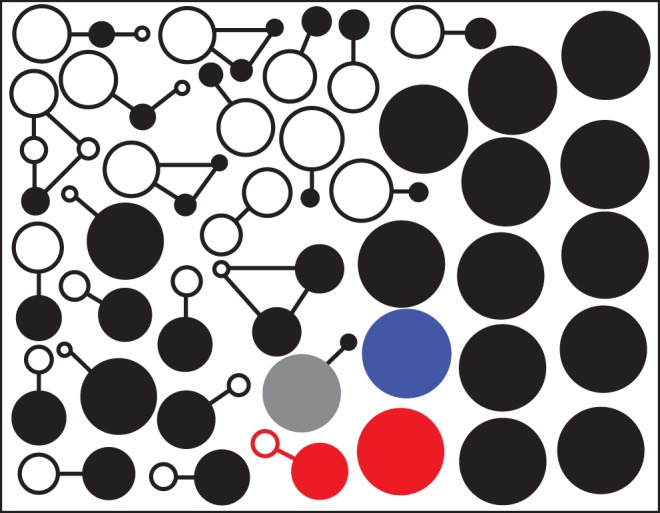
Genotypic diversity in 38 MRSA carriers. Each circle represents an MGE profile based on the presence or absence of four phages, two plasmids and the *qacA* gene. Connected circles are different MGE profiles from the same carrier. Circle size is proportional to MGE profile incidence in each patient and filled circles represent the MGE profile with the most phage and plasmid genes in that carrier. Black is lineage CC22, blue is lineage CC8, red is lineage CC45 and grey is lineage CC1. This figure appears in colour in the online version of *JAC* and in black and white in the print version of *JAC*.

The phenotypic differences between subisolates could be a reflection of the genetic variation caused by the acquisition or loss of MGEs such as plasmids, mediated by transducing phages. To further examine the samples, the genetic differences between the subisolates were analysed by PCR for four integrated prophage families, two plasmid (*rep*_10_ and *rep*_20_) families and *qacA*. Overall, 24 (63.2%) patients were colonized by genotypically different subisolates (Figure 3 and Table 1). The number of subisolates with genetic and AMR variation identified per patient ranged from one to eight (Table 1). In total, 64 different profiles were detected. The majority of patients had between one and four subisolates differing from the rest. Phenotypic variation in AMR correlated well with genetic differences.

Further analysis of phenotypic and genotypic subisolates showed each individual subisolate differed by only one or two AMR or MGE differences from other subisolates from the same patient. This included Patient 37 (Table 1), where eight variant profiles were identified; however, each variant differed from at least one other variant by only one AMR or MGE. These data are consistent with all variants in each patient originating from a single progenitor that has acquired or lost genes, rather than co-colonization with multiple MRSA CC22 introductions. The exception was Patient 22 (Table 1), who carried isolates from two MRSA lineages.

The most variation within nasal carriage populations was associated with the presence/absence of the *rep*_20_ gene in 50% of patients and variation in the presence of the *qacA* gene in 37.5% of patients. There was a strong positive correlation (Fisher's exact test, *P* < 0.0001) between susceptibility to erythromycin and lack of the *rep*_10_ gene. All subisolates susceptible to erythromycin were negative for the *rep*_10_ gene by PCR. Erythromycin resistance encoded by the *erm*(C) gene is often carried on a small non-conjugative plasmid with a *rep*_10_ replication locus.^3^

All subisolates had at least one prophage in the genome: 73% (552 out of 760) of subisolates were positive for Φ1, 84% (640) for Φ2, 82% (620) for Φ3 and 10% (78) for Φ6. Φ2 or Φ3 bacteriophage integrase gene was stable in all tested patients, while the other phages and plasmids showed evidence of instability. Comparison of swabs collected in 2012 showed less MGE diversity than those collected in 2013 (Fisher's exact test, *P* < 0.05).

The WGS analysis of five subisolates from Patient 19 (representing two AMR profiles and three MGE profiles) revealed extremely stable core genomes and no microvariations in the form of SNPs were detected. Differences between the subisolates in plasmids were consistent with AMR profile and PCR analysis. Specifically, two subisolates carried a 28 kb plasmid containing the *qacA* gene and showing an almost complete BLAST match to sequences deposited in the GenBank database as plasmid pSK1 (GU565967.1). Four subisolates carried a small 2.4 kb plasmid with *erm*(C) gene that matched 100% with plasmid CN1 (CP003981.1) (Figure S1).

### Bacteriophages from the swabs

Overall, 16 of 38 (42%) original nasal swabs were positive for the presence of lytic bacteriophages against *S. aureus* RN4220. The presence of bacteriophages did not correlate with phenotypic and genetic variation (Fisher's exact test, *P* = 1).

### Evidence of transducing ability of bacteriophages from the swabs

Φ19 and Φ20 and the control laboratory phage 80α were grown on erythromycin-resistant strain KS*ermB* in liquid culture and all produced phage titres of >10^4^ pfu/mL (Figure S2). Phage 80α produced a significantly higher value of phage titre (10^8^) compared with the phage titres of Φ19 and Φ20 (*t*-test, *P* < 0.01). When grown on 19A, the phage titres were lower (>10^6^) and, again, phage titres of Φ19 and Φ20 were significantly lower (>10^2^) (*t*-test, *P* < 0.05) (Figure S2). Control experiments without the addition of exogenous bacteriophages on KS*ermB* (prophage free) or 19A (carrying Φ1, Φ2 and Φ3 family prophages) resulted in a mean of 0 or 6 pfu/mL, respectively, which is significantly lower than when exogenous phages were added (*P* < 0.001). This indicates low levels of induction of the endogenous prophages found in the 19A genome under non-stressful conditions.

**Figure 4.**
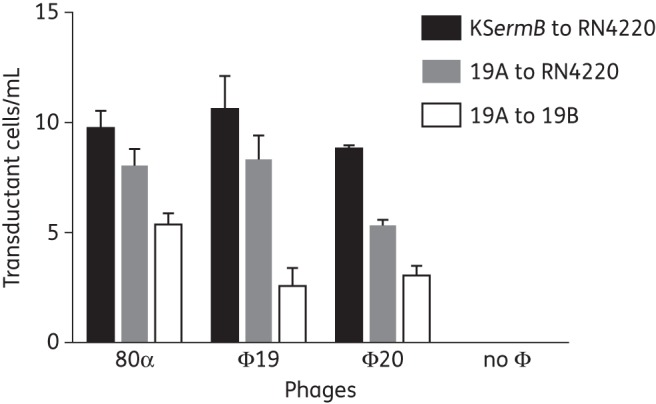
Transduction of resistance genes using phages isolated from nasal swabs. Phages 80α (control), Φ19 and Φ20 transduced erythromycin resistance genes from donors KS*ermB* or 19A [*erm*(C)] to recipients RN4220 or 19B. If no exogenous phages were added, no transduction was detected. Φ20 transduced resistance at a lower frequency than both 80α and Φ19 when strains were grown on 19A and resistance transferred to RN4220 (*t*-test, *P* < 0.001 and *P* < 0.01, respectively). 80α transduced resistance at a higher frequency than Φ19 and Φ20 when strains were grown on 19A and resistance transferred to 19B (*t*-test, *P* < 0.001). Transductant cells/mL of phage lysate are expressed as means of at least three different experiments with three replicates ± SD.

All of the tested phages (Φ19, Φ20 and 80α) were capable of generalized transduction of erythromycin resistance genes (Figure 4). Free bacteriophages Φ19 and Φ20 were equally capable of transducing erythromycin resistance genes from KS*ermB* into RN4220 strains compared with 80α phage, despite the lower lytic phage titre. High numbers of transductant cells were observed when phages 80α or Φ19 were multiplied on the 19A donor strain and used to transfer the *erm*(C) gene into RN4220. Free phage Φ20 was less efficient (*P* < 0.01) compared with phage 80α. Colonizing subisolate 19A was capable of acting as a donor and 19B was capable of acting as a recipient cell. All phages grown on 19A were capable of transferring the *erm*(C) gene into the 19B recipient strain; however, Φ19 and Φ20 were not as efficient as phage 80α (*P* < 0.001).

No statistically significant difference was found between the experiments with and without the addition of DNase (data not shown), indicating that transformation was not responsible for HGT. Both parents were negative by PCR for *tra* genes, which are necessary for conjugation. Therefore, HGT was likely due to bacteriophage-mediated transduction.

To confirm plasmid transfer, two progeny cells were compared by WGS analysis with the donor 19A and recipient 19B isolates. The donor strain and both progeny carried the 2.4 kb plasmid with the erythromycin resistance gene *erm*(C), which matched the CN1 plasmid (CP003981.1). Apart from the presence of the CN1 plasmid, both progeny had the same WGS as both the donor and the recipient strains. WGS data have been deposited in the European Nucleotide Archive under accession number PRJEB7630.

## Discussion

MRSA populations in the nose of human carriers varied in AMR profiles in 24% of colonized patients and the presence of MGEs in 63.2% of patients. Profiles varied despite most patients being colonized with a single MRSA clonal type. This suggests frequent HGT and loss of resistance genes and MGEs between isolates, both between patients^5,9,36^ and within patient populations. Recent studies also demonstrated an extremely high level of plasmid and phage transfer between isolates in an experimental piglet colonization model,^10^ even without antimicrobial selection. HGT of MGEs between *S. aureus* is thought to be a major contributor to the evolution, maintenance and success of new epidemic strains adapted to antimicrobials, new hosts and stresses.^37^

Generalized transduction is the most likely mechanism of high-level HGT in the nose.^4^ Free phages were detected in the nasal swabs of nearly half of the patients, which is substantially higher than previous reports.^31,38^ This may be due to the choice of *S. aureus* RN4220 as the host strain, as it is negative for known RM systems and therefore susceptible to lytic phage DNA that is normally restricted.^4^ It is possible a higher rate of lytic bacteriophages in the nose may be detectable with a broader range or more closely related host strains.^4,39^ Importantly, we have demonstrated that the free phages found in nasal swabs can be generalized transducing phages and therefore responsible for HGT.

Our results have implications for the identification of AMR in clinical samples. In 5/38 (13.2%) of patient swabs, the most prevalent AMR profile was not the most resistant (Figure 1). Therefore, the number of isolates chosen for AMR testing can greatly influence whether the full AMR profile present in the patient's sample is detected. Using mathematical modelling and the data from our patient population, we demonstrate that 18 colonies must be tested to detect all the resistances with 95% confidence. Thus, if only a few colonies from an MRSA carrier are tested, the presence of rare resistances can easily be missed. In this study, 20 subcolonies were experimentally tested and our mathematical analysis suggests there is a nearly 5% chance that some resistances were missed. Therefore, rare resistances might not have been sampled in our subisolate populations, leading to a possible overestimate of the probability of detecting all resistances in the specimen. Undersampling of colonies leads to underestimation of the reservoirs and incidence of resistance genes in MRSA populations.

Rare resistances may be more reliably detected if a sweep of colonies is sampled or if the specimen is plated onto selective agar for each antibiotic of interest. In the future, straight-from-sample molecular diagnostics may potentially identify rare resistances. If a patient is about to be prescribed a particular antibiotic, knowing whether there is a population of colonizing MRSA that could rapidly dominate may be clinically important. This study did not investigate other types of microbiology specimens. Chronic or contaminated samples where populations may have time to evolve may be more likely to have greater diversity, such as those colonizing the nose or cystic fibrosis lung,^17^ while samples from systemic acute infections are predicted to show less diversity due to bacterial population bottlenecks.^40^ Further studies are necessary.

MRSA epidemiological studies have suggested ‘clouds’ of diversity in nasal samples, based on WGS and SNP variation in the core genome.^14–16^ Here, we demonstrate that variation in AMR and MGEs was more prevalent than SNPs in human nasal populations, consistent with the piglet *S. aureus* colonization model.^10^ Epidemiological studies that rely on typing methods that include AMR or MGE profiles should consider this variation when interpreting data. WGS by next-generation sequencing methods that generate short reads and scaffold them onto reference genomes may not consider all the AMR and MGE data and may underestimate diversity.^37^ However, sequencing technologies that generate longer reads (e.g. Oxford Nanopore or PacBio) can potentially make extraction and analysis of MGEs and their AMR genes simpler. When used to construct epidemiological or phylogenetic relationships, these methods should consider the high level of MGE movement detected within patients. Overall, isolates with differing AMR and MGE profiles may be more related than previously considered and outbreak investigations involving nasal screening may have underestimated the prevalence of endogenous infection.

Our data suggested greater diversity in MGE profiles over time (2012 versus 2013), but AMR profiles did not show significant diversity over time. Since our 2012 patients were known to be negative for groin colonization and our 2013 patients' groin colonization was unknown, an alternative explanation is that patients colonized in multiple body sites carry more diverse populations in the nose. These findings require further investigation to swab multiple body sites and identify if particular MGEs are associated with these different colonizing sites.

A limitation of our study design is that we did not screen for loss of *mecA* within patients and cannot discount SCC*mec* is also unstable during colonization. A further limitation is that the free bacteriophages isolated from nasal samples may have been induced during the phage isolation process. If so, the phages were easily induced.

In conclusion, this study strongly suggests that genetic variation within nasal colonization populations is common. This is important because HGT can lead to the emergence of new isolates with enhanced resistance, adaptation to new hosts, immune evasion and pathogenic potential. Genome instability, possibly due to fitness costs and loss of MGEs, may also be common in MRSA nasal populations during colonization. An important consequence of extensive diversity within nasal populations is that sampling too few colonies from a specimen can lead to errors in AMR detection or identification of outbreak reservoirs.

## Funding

This work was supported by a studentship from the Medical Research Council (G0900205) and by St George's, University of London.

## Transparency declarations

J. A. L. has received fees from Pfizer for consultancy on *S. aureus* vaccines. All other authors: none to declare.

## Supplementary data

Tables S1 to S3 and Figures S1 and S2 are available as Supplementary data at *JAC* Online (http://jac.oxfordjournals.org/).

Supplementary Data
