# Supplementary material for: Within-host diversity of MRSA antimicrobial resistances
Source: J Antimicrob Chemother. 2015 May 8;70(8):2191–8. doi: 10.1093/jac/dkv119 (PMC4500776; doi:10.1093/jac/dkv119)
Supplement: Supplementary Data [file supp_70_8_2191__index.html]

Within-host diversity of MRSA antimicrobial resistances — Within-host diversity of MRSA antimicrobial resistances — Supplementary Data 

# Within-host diversity of MRSA antimicrobial resistances

## Supplementary Data

Supplementary Data

- Supplementary Data - Docx file
