# Supplementary material for: Within-host diversity of MRSA antimicrobial resistances
Source: J Antimicrob Chemother. 2015 May 8;70(8):2191–8. doi: 10.1093/jac/dkv119 (PMC4500776; doi:10.1093/jac/dkv119)
Supplement: Supplementary Data [file supp_dkv119_dkv119supp.docx]

**Supplementary data**

**Table S1. Phenotypic AMR profile in 38 MRSA carriers.** CC is clonal complex, and the unique profiles is the total number of different combinations of AMR phenotypes in each patient. This is different to Table 1 which shows total unique profiles of both AMR and MGE.

| patient no | CC | no of AMR profiles |
| --- | --- | --- |
|  |  |  |
| 37 | 22 | 2 |
| 34 | 22 | 2 |
| 36 | 22 | 2 |
| 25 | 22 | 1 |
| 19 | 22 | 2 |
| 20 | 22 | 2 |
| 29 | 22 | 1 |
| 38 | 22 | 2 |
| 12 | 22 | 1 |
| 10 | 22 | 1 |
| 18 | 22 | 1 |
| 24 | 22 | 1 |
| 30 | 22 | 1 |
| 27 | 22 | 1 |
| 28 | 22 | 1 |
| 31 | 22 | 2 |
| 16 | 22 | 1 |
| 6 | 22 | 1 |
| 32 | 22 | 1 |
| 17 | 22 | 1 |
| 11 | 22 | 1 |
| 26 | 22 | 1 |
| 3 | 22 | 1 |
| 2 | 22 | 1 |
| 15 | 22 | 1 |
| 1 | 22 | 1 |
| 23 | 22 | 1 |
| 14 | 22 | 1 |
| 4 | 22 | 1 |
| 5 | 22 | 1 |
| 9 | 22 | 1 |
| 8 | 22 | 1 |
| 13 | 22 | 1 |
| 7 | 22 | 1 |
| 22 | 1/22 | 2 |
| 33 | 45 | 1 |
| 35 | 45 | 3 |
| 21 | 8 | 1 |

**Table S2. Primers for detection of lineage, resistance genes and other MGEs.**

| **Target gene** | **Primer sequences**  **(5’ to 3’)** | **PCR fragment size (nt)** | **Annealing**  **temperature** | **Reference** |
| --- | --- | --- | --- | --- |
| *mecA* (Methicillin Resistance) | aaaccacccaatttgtctgc | 303 | 62°C | this study |
|  | tcaggttacggacaaggtga |  |  |  |
| *ermB* (Erythromycin Resistance) | AAAGGGCATTTAACGACGAA | 404 | 60°C | 41 |
|  | CTGTGGTATGGCGGGTAAGT |  |  |  |
| *ermC* (Erythromycin Resistance) | CGTAACTGCCATTGAAATAGACC | 235 | 62°C | 5 |
|  | agcaaactcgtattccacga |  |  |  |
| *rep10* | TGTTAGATATGATTGGCGGAA | 162 | 58°C | 42 |
|  | TTGGTCGTCGCCTCTCATTA |  |  |  |
| *rep20* | CCATCCAAAATGAATGGGTAG | 159 | 62°C | 42 |
|  | TAAGGCCAACACGTTCTTGA |  |  |  |
| Phage Integrase Φ1 | TTAAAGCTAAGTTCGGGCACAT | 780 | 60°C | 41 |
|  | TGTCAAAAATGGCTATTGCATC |  |  |  |
| Phage Integrase Φ2 | TTTGACTTTTCACGCGCTATTA | 777 | 60°C | 41 |
|  | ACACCGGCGATTTGATTATTAC |  |  |  |
| Phage Integrase Φ3 | TGAAAACACGTTGTTACGATGG | 780 | 60°C | 41 |
|  | ATCCGCCTTCTTTGAAAATGTA |  |  |  |
| Phage Integrase Φ6 | CCTTGAATTGATGGCGATTT | 203 | 60°C | 41 |
|  | TTGCTGGGGCTGTAGAAGTT |  |  |  |
| Phage Integrase Φ7 | TTCTGGCGCTTCCCTTTAAT | 500 | 60°C | 41 |
|  | AACACAGTCAAGCATACGCCT |  |  |  |
| *qacA* (quaternary ammonium resistance protein) | GCATTTTTAATTAATGTACCGTTTGC | 344 | 64°C | this study |
|  | GTGAAGCTAATAACAAAACAGATGC |  |  |  |
| *traA* (Conjugative plasmid) | cagcaacagcattgaaaggt | 219 | 62°C | 5 |
|  | ctatttgtcccgaggcgttt |  |  |  |
| AR22 (RM test 1) | AGGGTTTGAAGGCGAATGGG | 990 | 55°C | 43 |
|  | TCAGAGCTCAACAATGATGC |  |  |  |
| AR30 (RM test 1) | AGGGTTTGAAGGCGAATGGG | 203 | 55°C | 43 |
|  | CAACAGAATAATTTTTTAGTTC |  |  |  |
| AR45 (RM test 2) | AGGGTTTGAAGGCGAATGGG | 722 | 55°C | 43 |
|  | GGAGCATTATCTGGTGTTTTCC |  |  |  |
| AR1(RM test 2) | AGGGTTTGAAGGCGAATGGG | 1037 | 55°C | 43 |
|  | GGGTTGCTCCTTGCATCATA |  |  |  |
| BR8 (RM test 3) | CCCAAAGGTGGAAGTGAAAA | 680 | 55°C | 43 |
|  | CCAGTTGCACCATAGTAAGGGTA |  |  |  |
| BR5 (RM test 3) | CCCAAAGGTGGAAGTGAAAA | 1071 | 55°C | 43 |
|  | TCGTCCGACTTTTGAAGATTG |  |  |  |

**Table S3. Isolates and phages used in studies.**

| **strain/phage/plasmid** | **Relevant characteristic(s)** | **Source or reference** |
| --- | --- | --- |
| **Strains** |  |  |
| **19A** | Donor, *ermC*^+^, Φ1^+^, Φ2^+^ ,Φ3^+^ | Patient 19 sub-isolate |
| **19B** | Recipient, Φ1^+^, Φ2^+^ ,Φ3^+^ | Patient 19 sub-isolate |
| **RN4220** | Restriction-defective derivative of RN450, prophage negative | 44 |
| ***KSermB*** | RN4220 (pCN50::*-ermB-*) | This study |
| **Phages** |  |  |
| **Φ19** |  | patient 19 |
| **Φ20** |  | patient no 20 |
| **Φ80α** | Φ7^+^ | 23 |
| **Plasmid** |  |  |
| **pCN50** | Shuttle vector | 45 |
| **pCN50::*ermB*** | Em^r^ (*ermB*) ( N315) | This study |

**Figure S1. Diverse MGE profiles of MRSA sub-isolates from single patient.**

Figure shows five sequenced sub-isolates from patient no 19. The outermost ring (light blue) represents predicted genes in isolate HO 5096 0412 (CC22) used as a template. The innermost ring (black) shows genome of sub-isolate no 11, the orange sub-isolate no 2, the green sub-isolate no 9, the red sub-isolate no 1, the blue sub-isolate no 18. Genome of sub-isolates 2,9,1,18 were compared against the genome of sub-isolate 11 (the innermost ring –black) because this sub-isolate were positive for most of MGEs. Gaps in the rings represent MGEs which were present sub-isolate no 11 but absent in others. There was no variation in SNPs between all screened sub-isolates. Sub-isolates 1,2 and 18 were missing plasmid (gap highlighted in yellow) with *qacA* gene (gap highlighted in orange on yellow background), sub-isolate no 9 was missing plasmid with *ermC* gene (gap highlighted in orange).

**Figure S1**

sub-isolate no 11

sub-isolate no 2

sub-isolate no 9

sub-isolate no 1

sub-isolate no 18

plasmid with *qacA* gene

plasmid with *ermC* gene


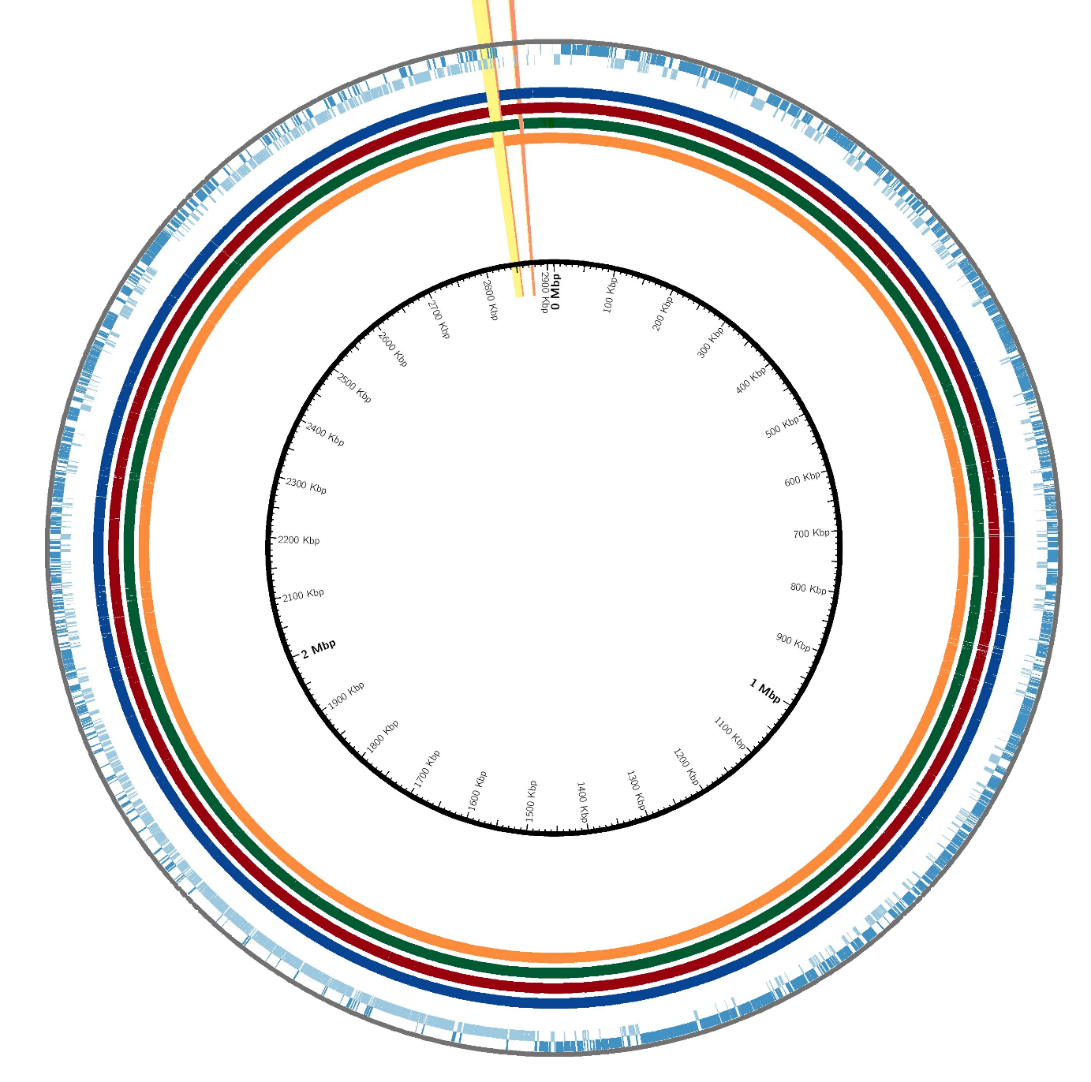


**Figure S2. Titration of lytic bacteriophages in lysates used for transduction.** Exogenous phages (80α, Φ19 and Φ20) were multiplied on *KSermB* or 19A (*ermC*). Plaque formation (pfu; plaques per ml of phage lysate ) were counted on the lawn of *S. aureus*  RN4220. Bars represent mean values of at least three experiments with three replicates ± standard deviation. 80α phage produced a significantly higher phage titer Φ19 and Φ20 when grown on KS*ermB* (t-test, p<0.01), and on 19A(t-test, p<0.05). Control experiments with no phage on *KSermB* or 19A resulted in a mean of zero or 6 pfu/ml respectively, significantly lower than when exogenous phages were added (p < 0.001).

**Figure S2**


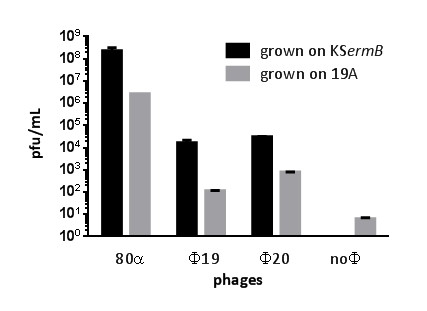


**Additional references**

41. Witney AA, Marsden GL, Holden MT *et al*. Design, validation, and application of a seven-strain Staphylococcus aureus PCR product microarray for comparative genomics. *Appl Environ Microbiol* 2005; **71**: 7504-14.

42. Jensen LB, Garcia-Migura L, Valenzuela AJ *et al*. A classification system for plasmids from enterococci and other Gram-positive bacteria. *J Microbiol Methods* 2010; **80**: 25-43.

43. Cockfield JD, Pathak S, Edgeworth JD *et al*. Rapid determination of hospital-acquired meticillin-resistant Staphylococcus aureus lineages. *J Med Microbiol* 2007; **56**: 614-9.

44. Kreiswirth BN, Löfdahl S, Betley MJ *et al*. The toxic shock syndrome exotoxin structural gene is not detectably transmitted by a prophage. *Nature* 1983; **305**: 709-12.

45. Charpentier E, Anton AI, Barry P *et al*. Novel cassette-based shuttle vector system for gram-positive bacteria. *Appl Environ Microbiol* 2004; **70**: 6076-85.
